# Case Report: Central nervous system relapse treated with elranatamab after prior BCMA and GPRC5D directed therapies in relapsed/refractory multiple myeloma

**DOI:** 10.3389/fonc.2026.1889721

**Published:** 2026-07-30

**Authors:** Raúl Pérez-Calle, Amalia Domingo-González, José Luis López-Lorenzo, Juan Fernando Bastidas-Tamayo, Begoña Gutiérrez-San José, Raquel Mata-Serna, Berta García-Corral, José Luis Santiago-Álvarez, Gala Vega-Achabal, Pilar Llamas-Sillero, Elham Askari

**Affiliations:** 1 Servicio Hematología Hospital Universitario Fundación Jiménez Díaz, Madrid, Spain; 2 Servicio Medicina Nuclear Hospital Universitario Fundación Jiménez Díaz, Madrid, Spain; 3 Servicio Radiología Hospital Universitario Fundación Jiménez Díaz, Madrid, Spain; 4 Servicio Análisis Clínicos Hospital Universitario Fundación Jiménez Díaz, Madrid, Spain; 5 Servicio Inmunología Hospital Universitario Fundación Jiménez Díaz, Madrid, Spain

**Keywords:** BCMA, bispecific Ab, CNS involvement, CNS relapse, elranatamab, multiple Myeloma

## Abstract

Central nervous system (CNS) involvement in multiple myeloma is a rare manifestation associated with aggressive disease biology and a particularly poor prognosis. With the advent of B-cell maturation antigen (BCMA)–directed therapies, evidence regarding their efficacy in CNS-MM remains limited. We report the case of a 70-year-old woman with relapsed/refractory MM who had previously been treated with proteasome inhibitors, immunomodulatory drugs, anti-CD38 monoclonal antibodies, an immune checkpoint inhibitor, an anti-BCMA antibody–drug conjugate, and a GPRC5D-directed bispecific antibody. She developed a late isolated CNS relapse 10 years after the initial diagnosis. Initial treatment for CNS involvement with pomalidomide, cyclophosphamide, and dexamethasone in combination with intrathecal chemotherapy achieved a transient response. Subsequently, treatment with elranatamab, a BCMA-directed bispecific antibody was initiated. After three cycles, cerebrospinal fluid analysis by flow cytometry demonstrated clearance of malignant plasma cells, with sustained negativity thereafter. The patient remains in CNS remission after 12 months of follow-up, with concordant clinical and radiological stability. This case highlights the feasibility and potential clinical activity of elranatamab in CNS-MM in a heavily pretreated patient with prior exposure to both BCMA and GPRC5D directed therapies.

## Introduction

Multiple myeloma (MM) accounts for approximately 1% of all malignancies and around 10% of hematologic neoplasms. Approximately 1–2% of MM patients present with extramedullary disease (EMD) at diagnosis, and up to 8% develop it during the course of the disease (1). The Central Nervous System (CNS) is one of the rarest extramedullary sites involved in relapsed or refractory (R/R) MM. It is also associated with aggressive biological features, including elevated LDH levels, plasmablastic morphology, circulating plasma cells, extramedullary localizations, and high-risk cytogenetic abnormalities (2). This condition carries a particularly poor prognosis, with published median overall survival (OS) ranging from 6 to 12 months (3).

In recent years, B-cell maturation antigen (BCMA)-directed therapies have transformed the therapeutic landscape of MM. Elranatamab is a humanized bispecific antibody targeting BCMA-expressing myeloma cells and CD3-expressing T cells. Despite the absence of robust data regarding central nervous system activity, its high overall response rate (ORR) in heavily pretreated patients and its off-the-shelf availability make it a potentially attractive option in this setting (2, 4).

We report a case of R/R MM with isolated CNS involvement treated with elranatamab in a patient previously exposed to both anti-BCMA antibody–drug conjugates and a GPRC5D-directed bispecific antibody, who achieved a clinical response and radiological and cerebrospinal fluid clearance of CNS disease as assessed by flow cytometry.

## Case report

A 60-year-old woman with no relevant medical history was diagnosed in 2013 with IgG kappa multiple myeloma. Initial laboratory evaluation revealed a serum monoclonal protein (M-protein) level of 3.3 g/dL, absence of detectable M-protein in urine, a kappa/lambda free light chain (FLC) ratio of 1300, and hypercalcemia. Magnetic resonance imaging (MRI) demonstrated bone lesions involving the C4, D7, and D9 vertebral bodies. Bone marrow aspiration showed 13% plasma cell infiltration by cytology. Fluorescence *in situ* hybridization (FISH) revealed monosomy 13 and t (4;14) translocation, while no TP53 mutations were detected by PCR. Based on these findings, a diagnosis of IgG kappa multiple myeloma, ISS stage II and R-ISS stage II, was established.

First-line treatment with bortezomib, cyclophosphamide, and dexamethasone (VCD) was administered for six cycles, followed by autologous hematopoietic stem cell transplantation (ASCT). The patient achieved a stringent complete response (sCR) and minimal residual disease (MRD) negativity in the bone marrow with a sensitivity of 10^-4^ measured by flow cytometry (**Figure 1**). Twenty-six months after ASCT, disease progression was detected by serum protein electrophoresis (SPEP), with asymptomatic bone relapse. Second-line treatment with bortezomib, thalidomide, and dexamethasone (VTD) was initiated. The patient received seven cycles, followed by maintenance therapy with quarterly bortezomib–dexamethasone, achieving a very good partial response (VGPR). Eighteen months later, biochemical progression occurred without clinical relapse. Given the patient’s preference for an all-oral regimen and access to ixazomib through a compassionate use program, third-line treatment with ixazomib, lenalidomide, and dexamethasone (IRD) was initiated, resulting in a partial response (PR).

**Figure 1:**
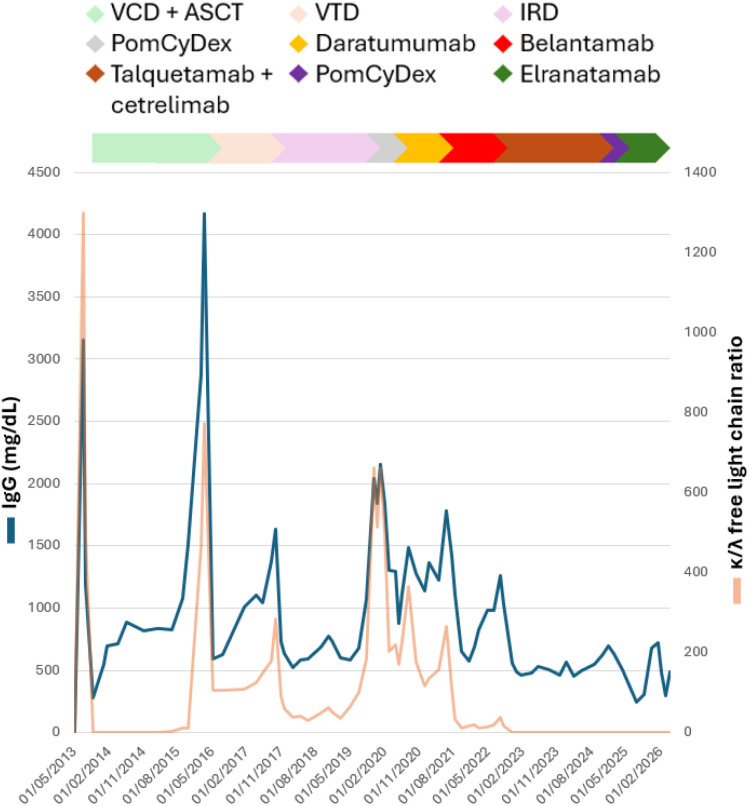
Longitudinal clinical and serological evolution of disease burden and treatment course in relapsed/refractory multiple myeloma. The graph depicts the temporal evolution of serum immunoglobulin G (IgG) levels (blue line, left y-axis; mg/dL) and the κ/λ free light chain (FLC) ratio (orange line, right y-axis) from initial diagnosis to the most recent follow-up. The x-axis represents chronological time. Fluctuations in both biomarkers reflect periods of response and disease progression across multiple lines of therapy. VCD, bortezomib, cyclophosphamide and dexamethasone; ASCT, Autologous Stem Cell Transplant; VTD, bortezomib, thalidomide and dexamethasone; IRD, ixazomib, lenalidomide and dexamethasone; PomCyDex, pomalidomide, cyclophosphamide and dexamethasone.

After two years, biochemical and medullary relapse was documented. Bone marrow evaluation demonstrated clonal plasma cell infiltration, and FISH revealed additional cytogenetic abnormalities, including 1q amplification and 17p deletion, consistent with high-risk cytogenetic features (5). Fourth-line treatment with pomalidomide, cyclophosphamide, and dexamethasone (PomCyDex) was initiated, but no clinical or biochemical response was observed. Fifth-line therapy with daratumumab, bortezomib, and dexamethasone (DVd) also failed to achieve hematologic response after nine months.

Given the lack of further response, sixth-line therapy with belantamab mafodotin was initiated on July 2, 2021. After two cycles, a partial response was achieved and maintained for 12 months, followed by biochemical progression. ^18^F-FDG PET/CT confirmed disease relapse with osseous involvement, showing new hypermetabolic lesions with increased FDG uptake in the frontal sinus, left maxillary sinus, left iliac bone, and right femoral diaphysis.

The patient subsequently received radiotherapy (30 Gy in 10 fractions) followed by systemic therapy. As seventh-line treatment, she was enrolled in a clinical trial and received talquetamab in combination with cetrelimab (an immune checkpoint inhibitor anti-PD-1 antibody) starting in October 2022. After 14 cycles, the patient achieved a complete response (CR), with complete metabolic response on ^18^F-FDG PET/CT. Treatment was discontinued in December 2023 due to gastrointestinal toxicity. .

In October 2024 (at 70 years of age), the patient developed lumbar pain radiating to both lower limbs, accompanied by bilateral distal paresthesias, progressive gait instability, and mild lower-limb weakness. An ^18^F-FDG PET/CT scan and spinal MRI performed in November 2024 revealed findings consistent with tumoral infiltration of the lumbosacral plexus, involving the right S1 nerve root and the left S1–S2 nerve roots, without evidence of parenchymal central nervous system involvement (**Figure 2**). Lumbar puncture demonstrated cerebrospinal fluid (CSF) infiltration by clonal plasma cells (26 cells/µL), exhibiting the following immunophenotype: CD138++ CD38+ CD56+ CD20− CD19− CD45±. No monoclonal protein was detected in serum or urine, and bone marrow evaluation confirmed MRD negativity at a sensitivity of 10^-4^.

**Figure 2:**
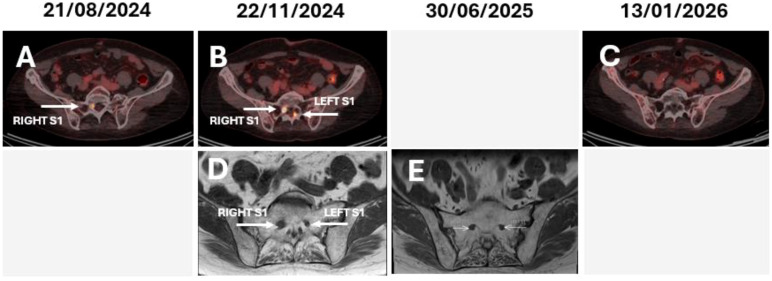
Multimodal imaging assessment of sacral involvement and treatment response. Axial fused PET/CT images **(A–C)**, and axial T1-weighted MRI sequences **(D, E)** at the level of S1 are shown. Follow-up PET/CT **(A)** demonstrates focal hypermetabolic activity in the right hemisacrum (S1), raising suspicion of disease involvement. Dedicated confirmatory imaging **(B)** reveals additional hypermetabolic uptake in the left hemisacrum, consistent with bilateral sacral involvement. Subsequent evaluation **(C)** shows absence of abnormal metabolic activity, indicating a favorable treatment response. Axial T1-weighted MRI at diagnosis **(D)** demonstrates bilateral sacral involvement with thickening of the S1 nerve roots, more pronounced on the right. Follow-up MRI at 7 months **(E)** shows resolution of nerve root thickening, supporting radiological response.

A diagnosis of symptomatic relapse with isolated CNS involvement was established. Treatment with local radiotherapy (RT) (25 Gy delivered in 5 daily fractions) was initiated, together with eighth-line therapy consisting of pomalidomide, cyclophosphamide, and dexamethasone (PomCyDex), as well as intrathecal (IT) chemotherapy (cytarabine 30 mg, methotrexate 12 mg, and prednisolone 10 mg) administered weekly as part of a therapeutic schedule. Baseline CSF analysis showed 26 cells/µL. After three IT administrations and two cycles of PomCyDex, a maximal response was achieved, with CSF cell counts decreasing to 0.4 cells/µL. Disease progression was documented three months after treatment initiation, with CSF malignant plasma cells increasing to 4.8 cells/µL, representing an increase of more than one log from the nadir. At that time, an additional IT dose was administered.

Elranatamab was initially administered on a weekly schedule; however, from cycle 5 onwards, dosing was switched to a monthly regimen due to prolonged grade 3 neutropenia attributed to treatment, together with recurrent grade 1–2 upper respiratory tract infections observed during the first cycles. The decision to reduce dosing intensity also took into account sustained disease control, as well as patient preference for reduced hospital dependence and logistical considerations. No other clinically relevant infectious complications were observed. Immunoglobulin replacement therapy was initiated as infection prophylaxis in the setting of IgG levels <400 mg/dL. No additional adverse events were reported, including immune effector cell–associated neurotoxicity syndrome (ICANS) or other neurological toxicities.

While receiving elranatamab, IT chemotherapy was only administered during follow-up lumbar punctures. The patient received IT therapy every 2-4 months for a total of 4 doses. The CSF analysis at the first lumbar puncture, 1 month after elranatamab initiation, showed a reduction in cell count to 1.2 cells/µL. CSF immunophenotyping became negative after the second lumbar puncture, approximately three months after initiation of elranatamab and remained negative thereafter (**Figure 3**).

**Figure 3:**
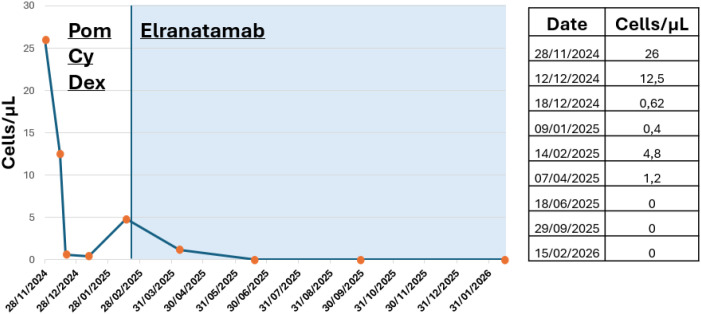
Longitudinal evolution of plasma cells in cerebrospinal fluid (CSF) assessed by multiparameter flow cytometry. Plasma cells were identified by the immunophenotype CD138++/CD38+/CD56+/CD20−/CD19−/CD45+/−. The left panel shows CSF plasma cell counts (cells/µL) over time across treatment phases with pomalidomide, cyclophosphamide, and dexamethasone (POM/Cy/Dex) followed by elranatamab. The right panel summarizes absolute counts at each sampling time point. A progressive decline is observed after elranatamab initiation, with complete clearance from June 2025 onwards (values of zero indicate undetectable disease within assay sensitivity). Orange dots indicate sampling time points and intrathecal chemotherapy administration.

Clinically, the patient experienced progressive neurological improvement following initiation of elranatamab. After a transient clinical improvement under treatment with PomCyDex, radiotherapy, and IT chemotherapy, gait instability, distal lower-limb paresthesias, fatigability, and a sensation of “blockage” in the legs recurred, with two falls occurring in February 2025. Within the first two months after initiation of elranatamab, lumbar pain and gait instability improved compared with baseline, with no further progression of weakness. Neurological examination showed partial motor recovery (dorsiflexion 4+/5, otherwise preserved strength), although distal sensory deficits and absent Achilles reflexes persisted. At the 6-month follow-up, the patient reported resolution of the previously described gait impairment and “blockage” sensation, with improved endurance and normalization of walking. Electromyography (EMG) demonstrated a stable distal sensory axonal polyneuropathy without significant changes over time, findings most likely related to prior neurotoxic therapies rather than active disease infiltration.

The last reassessment was performed in February 2026, 12 months after initiation of elranatamab. Lumbar puncture was negative for malignant cells, and bone marrow aspirate confirmed MRD negativity with a sensitivity of 10^-4^. Furthermore, 18F-FDG PET/CT performed in February 2026 showed no evidence of disease relapse, consistent with a sustained complete response.

## Discussion

CNS involvement in MM is a rare but severe manifestation, in R/R patients. It is estimated to occur in approximately 1–2% of MM cases during the disease course (3), with a reported median time to onset ranging from 12 to 43 months across published series (2). Recent studies have suggested an improvement in overall survival over the past decade, reaching approximately 12 months in patients treated from 2016 onwards (3). Although systemic therapy, alone or in combination with IT chemotherapy and/or radiotherapy, has improved outcomes, prognosis remains poor and no standard of care has been established for CNS involvement in MM.

A major therapeutic limitation in CNS-MM is the uncertainty regarding blood–brain barrier (BBB) permeability and CSF bioavailability of anti-myeloma agents, which significantly restricts treatment efficacy. A literature review has shown that immunomodulatory drugs (IMiDs) can cross BBB, although with variable permeability. Pomalidomide (CSF concentration 39%) and thalidomide (CSF concentration 30–60%) demonstrate higher BBB penetration than lenalidomide (CSF concentration 11%). Available evidence also supports CSF transferability of daratumumab; however, data on other monoclonal antibodies, such as isatuximab or elotuzumab, remain limited. Regarding proteasome inhibitors (PIs), bortezomib shows low CSF penetration (5%), carfilzomib has no detectable CSF transfer (0%), and marizomib shows approximately 30% CSF penetration (4).

In this context of limited CNS drug penetration and suboptimal activity of conventional agents, T-cell–redirecting immunotherapies have emerged as a promising therapeutic strategy. Although the use of BCMA-directed CAR T-cell therapy in patients with CNS-MM remains limited, emerging evidence is becoming available. *Wang* et al. (6) published a series of four patients, three of whom achieved a CR and one PR, with progression-free survival (PFS) of at least 9 months in one case. *Gaballa* et al. (7) reported a retrospective analysis of ten patients with CNS involvement, all of whom achieved a CR in CSF, with median PFS and OS of 6.3 and 13.3 months, respectively. In our case, BCMA-directed CAR T-cell therapy was declined due to prior exposure to belantamab, CNS involvement, and the absence of measurable disease in serum or urine.

In this challenging therapeutic context, a BCMA-directed bispecific antibody was considered the most appropriate available option. Elranatamab was therefore initiated following multidisciplinary discussion, based on its systemic activity in heavily pretreated R/R MM and the absence of alternative approved CNS-directed strategies.

Our case describes a patient with R/R MM who developed an isolated CNS relapse after seven prior lines of therapy. The patient had established risk factors for CNS involvement including prior EMD and high-risk cytogenetics. Notably, CNS relapse occurred more than 10 years after initial diagnosis, a considerably longer interval than that reported in most published series (3), and was confined exclusively to the CNS, a pattern observed in approximately 20–30% of CNS-MM cases (3, 10, 11). Importantly, the patient had been previously exposed to both an anti-BCMA antibody-drug conjugate and a GPRC5D-directed bispecific antibody, highlighting the heavily pretreated nature of the disease.

In this context, BCMA-directed bispecific antibodies represent a validated therapeutic option in triple-class refractory multiple myeloma. Elranatamab has demonstrated substantial efficacy in the pivotal MagnetisMM-3 trial, with an overall response rate of approximately 70% (8), including sustained activity in patients previously exposed to BCMA-targeted therapies, with an ORR of approximately 45% (9). However, data regarding BBB penetration and CNS activity remain lacking, and the biological basis of CNS efficacy is still unclear. Its activity in this setting may be partially explained by T-cell trafficking across a disrupted or inflammatory BBB, enabling local immune-mediated cytotoxicity, as described in other T-cell–mediated immunotherapies.

Despite these limitations, accumulating evidence suggests that CNS involvement should not preclude the use of T-cell–redirecting therapies, including bispecific antibody approaches, particularly within multimodal treatment strategies.

To date, only two cases of elranatamab use in CNS-MM have been reported. *Mutlu* et al. (12) described a 37-year-old patient with R/R MM with combined CNS and bone involvement treated with elranatamab in combination with daratumumab and dexamethasone for at least six cycles, achieving a clinical response. Grade 2 cytokine release syndrome (CRS) was successfully managed with tocilizumab. Similarly, *Uemura* et al. (13) reported a 55-year-old patient with isolated CNS relapse treated with elranatamab monotherapy who developed persistent grade 1 CRS requiring tocilizumab and dexamethasone, achieving CSF negativity after one month and maintaining clinical remission for at least six months. Although these cases suggest potential CNS activity of elranatamab, the evidence remains extremely limited and should be interpreted with caution.

A particularly relevant aspect of this case is the prior exposure to belantamab mafodotin, another BCMA-targeted agent. The patient received eight cycles, achieving a PR followed by disease progression after approximately one year of treatment. Elranatamab was administered four years later. Despite targeting the same antigen, the mechanisms of action differ substantially. Belantamab mafodotin specifically binds to BCMA, delivering monomethyl auristatin F (MMAF) into MM cells, and indirectly recruits immune system cells. On the other hand, elranatamab induces T-cell-mediated cytotoxicity through CD3 engagement. The different modes of action, along with the long period between the two drugs, are probably crucial in the subsequent response to treatment.

The interpretation of CNS response in this case is limited by the multimodal therapeutic approach. Prior to elranatamab, the patient received radiotherapy, IT chemotherapy, and PomCyDex, which resulted in only transient and incomplete clinical and cytological improvement. During this line of treatment, IT therapy was administered as part of an active scheduled strategy every week. During elranatamab treatment, IT treatment posology changed substantially. It was exclusively restricted to follow-up lumbar punctures, which were performed every 2-4 months Sustained complete remission was not achieved despite radiotherapy and intensive intrathecal chemotherapy. In contrast, complete and durable CSF remission was observed after initiation of elranatamab, even with a reduction in intrathecal treatment frequency and no further radiotherapy, supporting a dominant role of elranatamab in disease control, although a synergistic contribution of prior modalities cannot be excluded.

## Conclusion

To our knowledge, this is the first reported case of elranatamab use in CNS-MM in a patient previously exposed to an anti-BCMA antibody–drug conjugate and a GPRC5D-directed bispecific antibody. This case highlights the potential activity and favourable safety profile of elranatamab in CNS-MM, a setting with limited therapeutic options and poor prognosis. Importantly, the observed response supports the potential role of sequential BCMA targeting treatments. Further studies are warranted to define the CNS activity, efficacy, and safety of elranatamab in larger cohorts, as well as the biological mechanisms underlying CNS response.
